# Improved Production of Sublancin 168 Biosynthesized by *Bacillus subtilis* 168 Using Chemometric Methodology and Statistical Experimental Designs

**DOI:** 10.1155/2015/687915

**Published:** 2015-08-03

**Authors:** Shengyue Ji, Weili Li, Haiyun Xin, Shan Wang, Binyun Cao

**Affiliations:** College of Animal Science and Technology, Northwest A&F University, 22 Xinong Road, Yangling, Shaanxi 712100, China

## Abstract

Sublancin 168, as a distinct S-linked antimicrobial glycopeptide produced by *Bacillus subtilis* 168, is effective in killing specific microorganisms. However, the reported yield of sublancin 168 is at a low level of no more than 60 mg from 1 L fermentation culture of *B. subtilis* 168 by using the method in the literature. Thus optimization of fermentation condition for efficiently producing sublancin 168 is required. Here, Box-Behnken design was used to determine the optimal combination of three fermentation parameters, namely, corn powder, soybean meal, and temperature that were identified previously by Plackett-Burman design and the steepest ascent experiment. Subsequently, based on the response surface methodology, the quadratic regression model for optimally producing sublancin 168 was developed, and the optimal combination of culture parameters for maximum sublancin 168 production of 129.72 mg/L was determined as corn powder 28.49 g/L, soybean meal 22.99 g/L, and incubation temperature 30.8°C. The results showed that sublancin 168 production obtained experimentally was coincident with predicted value of 125.88 mg/L, and the developed model was proved to be adequate, and the aim of efficiently producing sublancin 168 was achieved.

## 1. Introduction

Sublancin 168 is a novel and distinct S-linked bacteriocin glycopeptide consisting of 37 amino acids and is produced by* Bacillus subtilis* 168 strains [1, 2]. Based on its potent antimicrobial activity in inhibiting special Gram-positive bacteria, including* B. megaterium*,* B. subtilis* 6633, the pathogenic microbes* Streptococcus pyogenes,* and* Staphylococcus aureus* [1], this antimicrobial peptide could be used in a wide range of commercial applications, such as agriculture, cosmetics, and pharmaceutical field. However, the host strain was cultured with a medium used for producing subtilin [1, 3], which contains (per liter) sucrose 20 g, citric acid 11.7 g, Na_2_SO_4_ 4 g, (NH_4_)_2_HPO_4_ 4.2 g, yeast extract 5 g, 100 mL of a salt mixture (KCl 7.62 g, MgCl_2_·6H_2_O 4.18 g, MnCl_2_·4H_2_O 0.543 g, FeCl_3_·6H_2_O 0.49 g, and ZnCl_2_ 0.208 g in 1000 mL H_2_O), and sufficient NH_4_OH to bring the pH to 6.8–6.9. As a result of complex medium ingredients and nonspecialized medium for producing sublancin 168, some events of the pinkish-brown color, fruity odor, and pH value near 6 that accompanying with good sublancin 168 production did not always occur, whereupon the production of sublancin 168 was usually at a low level of no more than 60 mg from 1 L bacterial culture [1]. The low yield of sublancin 168 has constrained its commercial application, and the optimization of fermentation conditions is required to allow for efficient production of sublancin 168.

Recently, there has been an increasing interest in response surface methodology processes for improving productivities of natural bioactive agents [4, 5]. Several bioactive proteins, such as eicosapentaenoic acid [6], tostadin [7], and antimicrobial compounds [8, 9], produced by bacteria strains optimized through response surface methodology have been recorded. However, literature is lacking cultivation optimization of sublancin 168 produced by* B. subtilis* 168 using chemometric and statistical methodology.

The aim of this current work was to efficiently produce sublancin 168 via optimizing the variables of medium compositions and culture conditions by using statistical tools in shake-flasks. In the first step, Plackett-Burman design as an effective technique was used to screen remarkable variables. Subsequently, the steepest ascent was utilized to approach the optimal region. At last, Box-Behnken design and response surface analysis were employed to ascertain the optimum levels of the factors which significantly effect sublancin 168 productions. In this study, the sublancin 168 production at a high level is achieved through adopting chemometric and statistical methodology.

## 2. Materials and Methods 

### 2.1. Materials

Yeast extract and tryptone were purchased from Difco (Detroit, USA). Corn powder and soybean meal were purchased from Sinopharm Chemical Reagent Beijing Co., Ltd. (China) and Shandong Litong Biotechnology Co., Ltd. (China), respectively, and were passed through 60-mesh sieve. Other chemicals used were of chemical grade.

### 2.2. Bacteria Strains and Fermentation Condition

*B. subtilis* 168 (ATCC 27370), the producer of sublancin 168, was utilized in the current work [1]. The strains were maintained on Luria-Bertani medium (LB) agar slant with the following composition (g/L): tryptone 10.0, yeast extract 5.0, NaCl 5.0, and agar 18.0 with the pH value to 7.0. After culturing at 37°C for 28 hours, the slants were subcultured once a month and stored at 4°C.

Seed culture of* B. subtilis* 168 was prepared by culturing bacterial strains in a 250 mL flask containing 50 mL LB liquid medium at 37°C, 225 rpm for 12 hours. Subsequently, 1 mL prepared seed culture was inoculated into 250 mL flask containing 50 mL culture medium (g/L): peptone 10.0, starch 15.0, KH_2_PO_4_ 4.0, and (NH_4_)_2_SO_4_ 4.0, and the pH was adjusted to 7.0, and furtherly cultured for 48 hours. After fermentation, the culture supernatant was harvested by removing the cells and the debris through centrifugation 10,000 g for 5 min. Each test was repeated three times and the average of sublancin 168 concentration was taken as the response.

### 2.3. Screen of Carbon and Nitrogen Sources

The optimal nitrogen and carbon sources effecting sublancin 168 production were screened by one variable at a time (OVAT) approach. The evaluations of different simple and complex nitrogen (yeast extract, peptone, soybean meal, urea, and (NH_4_)_2_SO_4_) and carbon sources (corn powder, glycerol, sucrose, lactose, starch, maltose, and glucose) on sublancin 168 production were performed one by one (Table 1). The above different nitrogen sources (5 g/L) and carbon sources (10 g/L) instead of peptone and starch were taken into the culture procedure as described above.

### 2.4. Plackett-Burman Design

The Plackett-Burman design is a powerful tool for rapidly screening and determining the important variables that has significant influence on the production response. This method was very useful for picking the most important factors from a long list of candidate factors [10]. In this work, different cultivation parameters (inoculum size, initial pH, incubation temperature, and incubation time) and medium components (peptone, soybean meal, starch, corn powder, KH_2_PO_4_, and (NH_4_)_2_SO_4_) were evaluated utilizing Plackett-Burman design to identify the important factors influencing sublancin 168 production greatly. Each factor at two levels was examined based on Plackett-Burman factorial design: −1 and +1 for low and high level, respectively [11]. On the preliminary study, it was found that the optimal temperature for producing sublancin 168 by* B. subtilis* 168 was at 32°C. Therefore, in Plackett-Burman experiment, the culture temperature test level was set between 30°C and 34°C.  Table 2 illustrates the factors under investigation and the levels of each factor setting in the experimental design. Response values were determined based on sublancin 168 productions.

The Plackett-Burman design was established by SAS software package (version 9.1.3, SAS Institute Inc., Cary, NC, USA) in terms of the following first-order model:

(1) $$\begin{array}{c} Y=\beta_{0}+\overset{k}{\underset{i=1}{\sum }}\beta_{i}X_{i}, \end{array}$$

where *Y* refers to the response (i.e., sublancin 168 production) and *β*_0_, *β*_*i*_, *X*_*i*_, and *k* represent the constant, the linear coefficient, the level of the independent variables, and the number of involved variables, respectively.

In addition to the variables of real interest, the Plackett-Burman design considers insignificant dummy variables, which are introduced to evaluate the experimental error and the variance of the first-order model. In this work, 10 variables were checked in 20 trials (Table 3). Every trial was performed three times, and the average sublancin 168 production was applied as the response variable. Regression analysis determined the variables that had a significant effect (*P* < 0.05) on sublancin 168 production, and these variables were subsequently evaluated in further optimization experiments.

### 2.5. Steepest Ascent Method

In general, some variations of the optimum culture condition for the system exist between the actual optimum and the initial estimate. In such case, the single steepest ascent experiment was performed to optimize the variables influenced sublancin 168 production significantly [12].

### 2.6. Response Surface Methodology

Through the Plackett-Burman design experiment, the significant variables were selected as follows: soybean meal, corn powder, and incubation temperature. After that, the Box-Behnken design, a type of response surface methodology, was used to determine the optimum level of these selected variables for producing sublancin 168 as highly as possible. With the help of the statistical software package “Design Expert 8.0.5b” (Shanghai TechMax Co., Ltd., Shanghai, China), the experimental design was analyzed and 15 experiments in all were formulated. The central values of every variable were coded 0. The maximum and minimum ranges of the variables were set up, and the whole experiment program in terms of their coded and actual values is shown in Table 5. In all trials the response values (*Y*) were the average of three replicates.

### 2.7. Batch Fermentation in a 5 L Bioreactor

To investigate the behaviour of sublancin 168 accumulation, batch fermentations were conducted in a 5 L bioreactor (NBS Co., USA). The prepared seed culture was inoculated (2%, v/v) into the optimal medium with an initial pH 7.0. According to the preexperiment results (data not shown), the bioreactor was operated with optimized temperature, airflow at 1.5 vvm, and stirring at 500 rpm, and the pH was uncontrolled during fermentation.

### 2.8. Quantitative Determination of Sublancin 168 Content

Isolation and purification of sublancin 168 were carried out as previously described [1], with slight modification. The collected supernatant was made in 1 M NaCl and subjected to a hydrophobic interaction chromatography of 25 mL Toyopearl Butyl-650 column (Tosoh, Tokyo, Japan), and then a solution of 50 mM NaAc, pH 4.0, was used to wash down the sublancin. Subsequently the elution was made in 0.1 trifluoroacetic acid (TFA) and subjected to a semipreparative Zorbax 300SB-C8 column (250 × 9.4 mm, 5 *μ*m particle size, 300 Å pore size) (Agilent, Englewood, CO) with a linear 0–60% acetonitrile gradient at a flow rate of 1.0 mL/min. The active fractions were collected and applied to an analytical Zorbax 300SB-C8 column (150 × 4.6 mm, 5 *μ*m particle size, 300 Å pore size) (Agilent, Englewood, CO) with the same conditions as the first step. The absorbances at 214 nm, 254 nm, and 280 nm were monitored. The concentration of purified sublancin 168 was determined by UV spectrophotometry [13, 14]. Using purified sublancin as standard sample, the fermentation broths were applied to analytical Zorbax 300SB-C8 column to determine sublancin 168 concentrations with the method used in purification of sublancin 168.

### 2.9. Statistics

During this study, each experiment was repeated three times, and all of the data were expressed as means ± standard deviations.

## 3. Results and Discussion

### 3.1. Screening Optimal Carbon Sources and Nitrogen Sources

According to the fermentation result (data not shown) obtained by using the method reported in the literatures [1, 3], there is a no more than 60 mg sublancin 168 from one liter bacterial culture. Thus, a new fermentation method with some different media and culture conditions is required to efficiently produce sublancin 168. As illustrated in Table 1, among the evaluations with different nitrogen sources, soybean meal showed an outstanding effect on the sublancin 168 production of 58.40 mg/L, followed by peptone of 50.11 mg/L. Urea had played an insignificant role on this peptide production. Among the different tested carbon sources, corn powder had a prominent effect on the sublancin 168 production of 67.66 mg/L, followed by starch of 50.65 mg/L, and glycerol had a sight effect on the yield of sublancin 168. Corn powder and soybean meal play an important role in production improvement of interest products, such as* Acinetobacter *sp. DNS_32_ strain [15],* B. subtilis* WHK-Z12 spore [16], *β*-glucanase from* B. subtilis* ZJF-1A5 [17], and alkaline protease from* Bacillus* sp. RKY3 [18]. In this study, the results of screening optimal carbon sources and nitrogen sources suggested that corn powder and soybean meal were also important for* B. subtilis* 168 strains to efficiently produce sublancin 168.

### 3.2. Plackett-Burman Design

**Figure 1:**
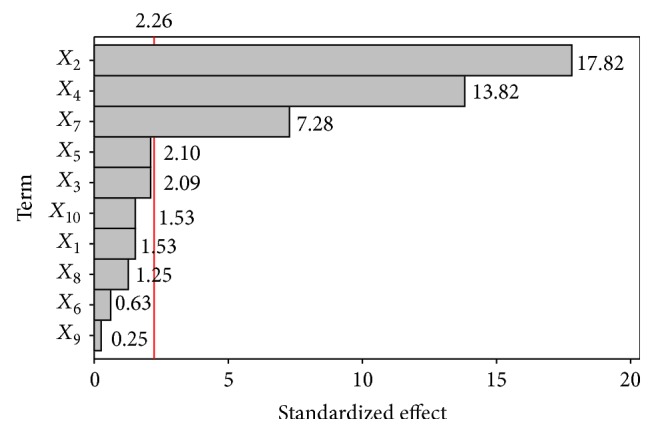
Pareto chart of standardized effects on sublancin 168 production. *X*_1_, *X*_2_, *X*_3_, *X*_4_, *X*_5_, *X*_6_, *X*_7_, *X*_8_, *X*_9_, and *X*_10_ are the coded values of peptone, corn powder, starch, soybean meal, KH_2_PO_4_, (NH_4_)_2_SO_4, _incubation temperature, initial pH, incubation time, and inoculum size, respectively. The chart has a vertical line (i.e., standardized effect = 1.886) at the critical *t*-value for *α* of 0.20. The bars are shown in order of the size of the effects, and the standardized effect of every term was displayed on the top of its corresponding bar.

The Plackett-Burman design was employed to evaluate the relative importance of cultivation parameters and different medium components (Figure 1). The main effect of each variable upon sublancin 168 production was evaluated as the difference made between both averages of measurements at the high level (+1) and at the low level (−1) correspondingly (Table 2). The data in Table 3 showed a wide variation from 60.31 mg/L to 121.38 mg/L. The results suggested that higher productivity of sublancin 168 was achieved from medium optimization. The significant variables affecting sublancin 168 productivity are soybean meal, corn powder, and incubation temperature from the calculation of* t*-values and *P* values (Table 2 and Figure 1). The incubation time from 28 h to 40 h and inoculum size from 1% to 3% play an insignificant role on sublancin 168 production.

Based on (1) and analyzed by using Minitab, a first-order model was fitted to the results obtained from the twenty experiments as the following equation:

(2) Y mg/L=95.56+1.17X1+13.60X2+1.60X3+10.54X4+1.60X5−0.48X6+5.55X7−0.96X8+0.19X9−1.17X10,

where *Y* is the sublancin 168 production and *X*_1_, *X*_2_, *X*_3_, *X*_4_, *X*_5_, *X*_6_, *X*_7_, *X*_8_, *X*_9_, and *X*_10_ are the coded values of peptone, corn powder, starch, soybean meal, KH_2_PO_4_, (NH_4_)_2_SO_4,_ incubation temperature, initial pH, incubation time, and inoculum size, respectively. The goodness of the regression model was determined by the coefficient of determination *R* ^2^ whose value is 96.76% and suggests that only 3.24% of the total variation could not be explained by the model. Hence it was reasonable to take the regression model to analyse the tread in the response.

### 3.3. Steepest Ascent

Even though Plackett-Burman design allows for the rapid selection of the significant variables affecting productivity of sublancin 168, the optimum levels of the variables cannot be predicted by this method. The method of steepest ascent is a procedure for moving sequentially along the path of steepest ascent and in the direction of the maximum increase in the response. In order to move the variables rapidly to the general vicinity of the optimum levels, the path of steepest ascent was used to find the proper direction to change the variables by increasing the incubation temperature and the concentration of soybean meal and corn powder to improve the production of sublancin 168. The results showed that the sublancin 168 production reached a yield plateau during the fifth step (Table 4). Thus, these three variables were selected for further optimization.

As illustrated in Table 4, the sublancin 168 production did not further increase with the increase of concentrations of corn powder and soybean meal and the increase of temperature. For corn powder and soybean meal, the yield of sublancin 168 decreased from 122.6 mg/L to 109.0 mg/L when the concentrations of corn powder and soybean meal increased from 28 g/L to 32 g/L and from 24 g/L to 28 g/L, respectively. The increase temperature from 33°C to 35°C may make the cells more difficult to biosynthesize sublancin 168; therefore, the sublancin production was not improved.

### 3.4. Box-Behnken Design

To determine the optimum levels of these important independent variables (soybean meal, corn powder, and incubation temperature) according to the above results, a 3-factor Box-Behnken design with 3 levels involving 3 replicates at center point was introduced to fit a second-order response surface. Table 5 shows the design matrix and the corresponding experimental data. The quadratic regression equations were obtained according to sublancin 168 production after the above results were analysed through standard analysis of variance (ANOVA). With the data of designed experiments, the polynomial regression model (in coded value) for sublancin 168 yield *Y* was regressed only with respect to the significant factors and was shown as follows:

(3) Y=124.15+5.07X1−5.51X2−5.63X3+2.06X1X2+3.65X1X3+13.47X2X3−21.51X12−18.35X22−14.91X32,

where *Y* predicates the sublancin 168 production, *X*_1_ is corn powder, *X*_2_ is soybean meal, and *X*_3_ is incubation temperature.

Based on *F*-test and ANOVA using the SAS software package, the statistical significance of (3) was evaluated. As shown in Table 6, *F*-value of the model is 2507.25, and *F*-value for lack of fit is 3.92. The high *F*-value and nonsignificant lack of fit indicate that the model (3) is a good fit. This result indicates that the model used to fit response variable is significant (*P* < 0.0001) and adequate to represent the relationship between the responses and the independent variables. And the ANOVA (*F*-test) for this work is shown in Table 7. The value of determination coefficient *R* ^2^ is 0.9996, which means that we are able to explain 99.98% of results for sublancin 168 production using the calculated model. This result indicates that the model used to fit response variable is significant (*P* < 0.0001) and adequate to represent the relationship between the responses and the independent variables. Meantime, the significance of the model was satisfactorily confirmed by the adjusted determination coefficient (*R*_Adj_^2^ = 0.9993) and predicated determination coefficient (*R*_pre_^2^ = 0.9998). Thus using this model to estimate the response trends is considered to be reasonable.

The model coefficient calculated from the regression analysis for each significant variable is shown in Table 7. Table 7 shows that the regression coefficients of individual linear, quadratic terms, and two cross products are statistically significant at 95% confidence level.

**Figure 2:**
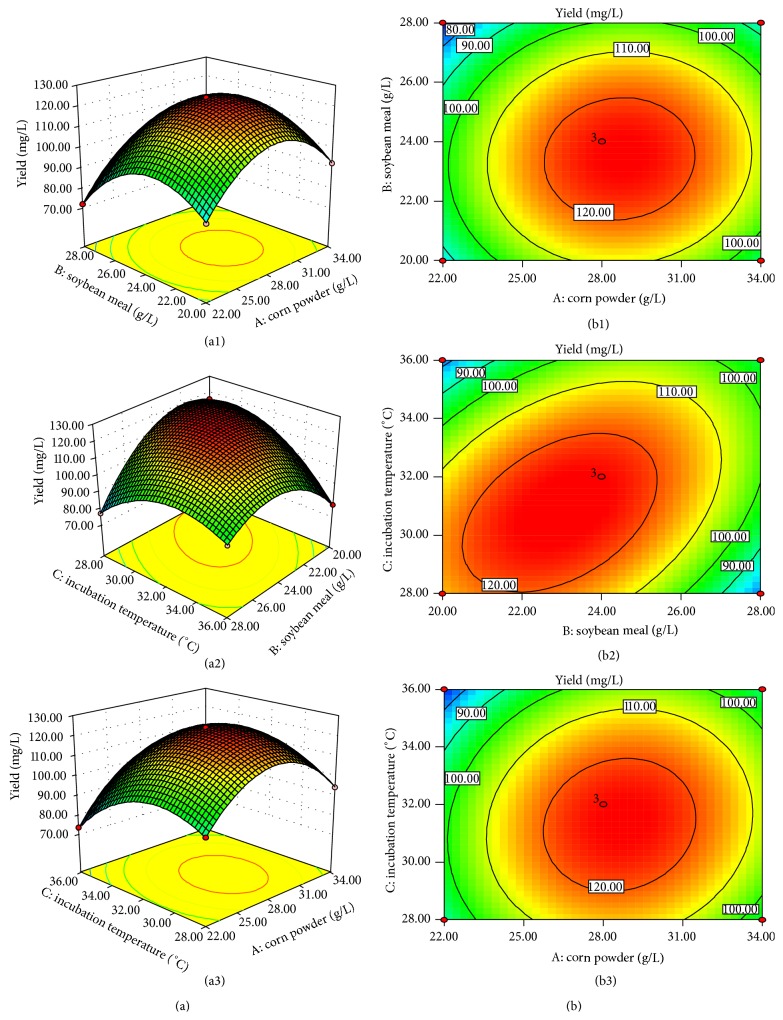
3D response surface curves (a) and 2D contour plots (b) predicting for sublancin 168 production by* Bacillus subtilis* 168 through optimization of variables. The interaction between (a1, b1) soybean meal and corn powder, (a2, b2) incubation temperature, and soybean meal and (a3, b3) incubation temperature and corn powder.

Three-dimensional (3D) response surface plots (Figure 2(a)) and two-dimensional (2D) contour plots (Figure 2(b)) are the graphical representations of the quadratic polynomial regression equation and usually illustrate the relationships between the experimental levels of each variable and corresponding response. The sublancin 168 production is shown in Figure 2 by 3D response surface plots and their respective 2D contour plots. Each figure reveals the interaction of two variables meanwhile the other is kept at zero level. In the 3D response surface plots and 2D contour plots as shown, the interaction exists within every pair of selected three factors and the effects are significant.

The model reveals that the corn powder concentration (*X*_1_), soybean meal concentration (*X*_2_), and temperature (*X*_3_) had a significant effect (*P* < 0.0001) on the sublancin 168 production (*Y*). Positive coefficient of *X*_1_ indicated a linear effect to increase, and negative coefficient of *X*_2_ and *X*_3_ suggested a linear effect to decrease. Meanwhile, quadratic term *X*_1_^2^, *X*_2_^2^, and *X*_3_^2^ had the negative effect. The graphs (Figure 2) illustrate the changes in the parameter modelled as the two factors move along those levels, while the other factor held constant at the central point. According to (3), it was predicted that a maximum sublancin 168 production of 125.88 mg/L appeared at 22.99 g/L soybean meal and 28.49 g/L corn powder, while temperature was held at 30.8°C.

### 3.5. Model Verification in Shake-Flask

The availability of the regression model of the sublancin 168 production using the calculated optimal medium compositions and culture condition, namely, 22.99 g/L soybean meal, 28.49 g/L corn powder, and temperature at 30.8°C, was validated with triplicate experiments. The mean maximal value of sublancin 168 production was 129.72 mg/L, which agreed with the predicted value (125.88 mg/L) well. As a result, the model was considered to be accurate and reliable for predicting the sublancin 168 production by* B. subtilis* 168. However, there is a certain gap between the yield and the theoretical value, which may be caused by some factors other than the medium that affect the yield of sublancin 168 but not investigated in this work. In this study, the yield of sublancin 168 by* B. subtilis* 168 was improved from low level of no more than 60 mg/L up to 129.72 mg/L in optimized medium.

### 3.6. Validation of the Model in Bioreactor

Using the optimal medium and temperature, sublancin 168 reached repeatable yield of 135.4 mg/L in bioreactor batch fermentation after about 48 h of cultivation. Although the temperature and medium components were coincident in flask and bioreactor fermentations, the yield of sublancin 168 in bioreactor fermentation (135.4 mg/L) was higher than that in the shake-flask culture (129.72 mg/L), probably mainly due to the differences of aeration conditions. Even so, the working conditions of the bioreactor require further optimizations in future experiments to furtherly improve sublancin yield.

### 3.7. Influence of Corn Powder and Soybean Meal on Sublancin 168 Production

In* B. subtilis* 168, there are six proteins (SunI, SunA, SunT, BdbA, SunS, and BdbB) [2] taking part in biosynthesizing mature sublancin 168, and the biosynthesis of sublancin 168 is controlled under a complex regulatory network that involves a minimum of five transcriptional regulators, including Abh, AbrB, Rok, YvrG, and YvrH [19–21]. Corn powder and soybean meal are commonly substrates used by bacteria to produce enzymes and other secondary metabolites through fermentation.* B. subtilis* 168 possesses an ATP-dependent protein kinase which can be activated by several metabolites (fructose 1,6-diphosphate, gluconate-6-P, and 2-phosphoglycerate) when growing in the presence of corn powder. The activated protein kinase phosphorylates a seryl residue (ser-46) of HPr, a small phosphocarrier protein [22]. HPr probably have a direct or indirect control function on the complex regulatory network that regulates the biosynthesis of sublancin 168. Soybean meal is a highly concentrated source of protein [23] and can provide an excellent profile of amino acids for producing sublancin 168.

## 4. Conclusions

As a summary, response surface methodology combined with Plackett-Burman design and steepest ascent enabled us to optimize the sublancin 168 yield produced by* B. subtilis* 168. The optimal combinations of culture parameters for maximum production of sublancin 168 were determined as corn powder 28.49 g/L, soybean meal 22.99 g/L, and incubation temperature 30.8°C, and the maximum production of sublancin 168 was significantly improved from no more than 60 mg/L before optimization up to 129.72 mg/L. To our knowledge, this is first report of statistical optimization for sublancin 168 production, which would provide some important parameters for large scale fermentation of this agent.

## Figures and Tables

**Table 1:** Effects of different carbon sources and nitrogen sources on the yield of sublancin 168.

Carbon sources		Nitrogen sources	
Sources	Yield (mg/L)	Sources	Yield (mg/L)
Corn powder	67.66 ± 3.56	Yeast extract	28.89 ± 4.72
Glycerol	28.96 ± 4.39	Peptone	50.11 ± 4.56
Sucrose	30.55 ± 4.90	Soybean meal	58.40 ± 5.33
Lactose	29.61 ± 5.03	Urea	8.72 ± 3.18
Starch	50.65 ± 4.85	(NH_4_)_2_SO_4_	32.93 ± 2.10
Maltose	28.26 ± 5.10		
Glucose	21.40 ± 5.49		

Each experiment was repeated three times, and all of the data were expressed as means ± standard deviations.

**Table 2:** Variables and test levels for Plackett-Burman experiment.

Number	Variables	Code levels		Estimate	*t*-value	*P* value	Significance
		−1	1				
*X*_1_	Peptone (g/L)	8	12	2.33	1.01	0.3851	
*X*_2_	Corn powder (g/L)	20	30	27.20	11.83	0.0013	∗
*X*_3_	Starch (g/L)	10	20	3.19	1.39	0.2594	
*X*_4_	Soybean meal (g/L)	24	36	21.09	9.17	0.0027	∗
*X*_5_	KH_2_PO_4_ (g/L)	3	6	3.21	1.39	0.2575	
*X*_6_	(NH_4_)_2_SO_4_ (g/L)	3	6	−0.96	−0.42	0.7038	
*X*_7_	Incubation temperature (°C)	28	34	11.11	4.83	0.0169	∗
*X*_8_	Initial pH	6.5	8.5	−1.91	−0.83	0.0466	
*X*_9_	Incubation time (h)	28	40	0.38	0.17	0.8786	
*X*_10_	Inoculum size (%)	1	3	−2.34	−1.01	0.3834	

∗ indicates model terms are significant.

**Table 3:** Experimental design and results of the Plackett-Burman design.

Trials	Variable levels										Yield (mg/L)	
	*X*_1_	*X*_2_	*X*_3_	*X*_4_	*X*_5_	*X*_6_	*X*_7_	*X*_8_	*X*_9_	*X*_10_	Observed	Predicted
1	1	−1	1	1	−1	−1	−1	−1	1	−1	88.38 ± 3.84	90.91
2	1	1	−1	1	1	−1	−1	−1	−1	1	117.96 ± 2.37	115.41
3	−1	1	1	−1	1	1	−1	−1	−1	−1	97.42 ± 6.78	96.55
4	−1	−1	1	1	−1	1	1	−1	−1	−1	101.79 ± 3.41	98.33
5	1	−1	−1	1	1	−1	1	1	−1	−1	102.43 ± 3.74	99.73
6	1	1	−1	−1	1	1	−1	1	1	−1	92.45 ± 3.96	94.17
7	1	1	1	−1	−1	1	1	−1	1	1	107.47 ± 9.89	104.83
8	1	1	1	1	−1	−1	1	1	−1	1	121.38 ± 2.26	124.57
9	−1	1	1	1	1	−1	−1	1	1	−1	119.62 ± 2.27	117.05
10	1	−1	1	1	1	1	−1	−1	1	1	88.76 ± 5.85	90.81
11	−1	1	−1	1	1	1	1	−1	−1	1	119.63 ± 5.56	123.21
12	1	−1	1	−1	1	1	1	1	−1	−1	79.81 ± 4.77	80.87
13	−1	1	−1	1	−1	1	1	1	1	−1	118.85 ± 5.84	120.81
14	−1	−1	1	−1	1	−1	1	1	1	1	76.33 ± 3.15	77.53
15	−1	−1	−1	1	−1	1	−1	1	1	1	82.21 ± 2.79	80.17
16	−1	−1	−1	−1	1	−1	1	−1	1	1	77.19 ± 4.41	76.27
17	1	−1	−1	−1	−1	1	−1	1	−1	1	62.37 ± 5.76	61.05
18	1	1	−1	−1	−1	−1	1	−1	1	−1	106.22 ± 7.25	104.95
19	−1	1	1	−1	−1	−1	−1	1	−1	1	90.56 ± 2.95	90.05
20	−1	−1	−1	−1	−1	−1	−1	−1	−1	−1	60.31 ± 3.20	63.93

Each experiment was repeated three times, and all of the data were expressed as means ± standard deviations. *X*_1_, *X*_2_, *X*_3_, *X*_4_, *X*_5_, *X*_6_, *X*_7_, *X*_8_, *X*_9_, and *X*_10_ represent peptone (g/L), corn powder (g/L), starch (g/L), soybean meal (g/L), KH_2_PO_4_ (g/L), (NH_4_)_2_SO_4_ (g/L)_, _incubation temperature (°C), initial pH, incubation time (h), and inoculum size (%).

**Table 4:** Experimental design and corresponding response of steepest ascent.

Experiment number	Corn powder (g/L)	Soybean meal (g/L)	Incubation temperature (°C)	Yield (mg/L)
0	12	8	25	72.7 ± 2.97
0 + 1Δ	16	12	27	80.3 ± 4.11
0 + 2Δ	20	16	29	89.5 ± 2.42
0 + 3Δ	24	20	31	117.5 ± 3.58
0 + 4Δ	28	24	33	122.6 ± 1.64
0 + 5Δ	32	28	35	109.0 ± 4.17

Each experiment was repeated three times, and all of the data were expressed as means ± standard deviations.

**Table 5:** Experimental design and results of Box-Behnken optimization experiment.

Trials	*X*_1_	*X*_2_	*X*_3_	Yield (mg/L)	
				Observed	Predicted
1	22.00	24.00	36.00	73.66 ± 1.62	73.43
2	34.00	28.00	32.00	86.35 ± 2.78	86.78
3	28.00	28.00	28.00	77.37 ± 1.83	77.57
4	28.00	24.00	32.00	124.39 ± 1.92	124.15
5	22.00	28.00	32.00	71.97 ± 1.26	71.81
6	34.00	24.00	28.00	94.52 ± 3.73	94.75
7	28.00	28.00	36.00	92.97 ± 5.06	93.37
8	28.00	20.00	28.00	115.92 ± 2.90	115.53
9	22.00	20.00	32.00	86.35 ± 2.72	85.93
10	28.00	24.00	32.00	124.22 ± 1.24	124.15
11	28.00	24.00	32.00	123.85 ± 1.63	124.15
12	34.00	20.00	32.00	92.67 ± 3.56	93.37
13	34.00	24.00	36.00	90.80 ± 2.90	90.82
14	22.00	24.00	28.00	91.98 ± 3.72	91.95
15	28.00	20.00	36.00	77.48 ± 2.35	77.28

Each experiment was repeated three times, and all of the data were expressed as means ± standard deviations. *X*_1_, *X*_2_, and *X*_3_ represent corn powder (g/L), soybean meal, and incubation temperature (°C), respectively.

**Table 6:** Analysis of variances of the quadratic polynomial model.

Source	SS	DF	MS	*F*-value	*P* > *F*
Model	4776.00	9	530.67	2507.25	<0.0001
Lack of fit	0.90	3	0.30	3.92	0.2100
Pure error	0.15	2	0.08		

Total	4777.05	14			

*R* ^2^ = 0.9998, *R*_Adj_^2^ = 0.9993, and *R*_Pre_^2^ = 0.9998. SS: sum of squares; DF: degrees of freedom; MS: mean square.

**Table 7:** Results of regression analysis of the second-order polynomial model.

Factor	Coefficient estimate	Standard error	*F*-value	*P* > *F*
Intercept	124.15	0.27	2507.25	<0.0001
*X*_1_	5.05	0.16	963.01	<0.0001
*X*_2_	−5.47	0.16	1130.53	<0.0001
*X*_3_	−5.61	0.16	1189.84	<0.0001
*X*_1_ *X*_2_	2.02	0.23	76.82	0.0003
*X*_1_ *X*_3_	3.65	0.23	251.72	<0.0001
*X*_2_ *X*_3_	13.51	0.23	3449.98	<0.0001
*X*_1_^2^	−21.51	0.24	8068.33	<0.0001
*X*_2_^2^	−18.31	0.24	5847.41	<0.0001
*X*_3_^2^	−14.91	0.24	3877.87	<0.0001
